# Tobacco smoking and prevalence of communicable and non-communicable diseases in rural South Africa: A cross-sectional study

**DOI:** 10.21203/rs.3.rs-2730894/v1

**Published:** 2023-04-07

**Authors:** Glory Chidumwa, Stephen Olivier, Hloniphile Ngubane, Thando Zulu, Ronel Sewpaul, Gina Kruse, Nancy A. Rigotti, Mark J. Siedner, Krishna P. Reddy, Emily B. Wong

**Affiliations:** University of the Witwatersrand; Africa Health Research Institute, KwaZulu-Natal; Africa Health Research Institute, KwaZulu-Natal; Africa Health Research Institute, KwaZulu-Natal; Human Sciences Research Council; Massachusetts General Hospital; Massachusetts General Hospital; Africa Health Research Institute, KwaZulu-Natal; Massachusetts General Hospital; Africa Health Research Institute, KwaZulu-Natal

**Keywords:** Tobacco, smoking, communicable diseases, non-communicable diseases

## Abstract

**Background:**

South Africa is facing a convergence of communicable diseases (CDs) and non-communicable diseases (NCDs). The contribution of tobacco use to the burden of these conditions is unknown.

**Methods:**

We analyzed the associations between current tobacco smoking and four important CDs and NCDs in Vukuzazi, a cross-sectional study of individuals aged 15 years and older conducted between 2018–2020 in a demographic surveillance area in KwaZulu-Natal, South Africa. Data on HIV, active tuberculosis (TB), hypertension and diabetes mellitus were collected via direct measurement from participants.

**Results:**

Of 18,024 participants (68% female, median age 37 years [interquartile rage 23–56 years]), 1,301 (7.2%) reported current smoking. Prevalence of HIV infection was similarly high among people who currently smoked (34.6%) and people who had never smoked (33.9%). However, among people living with HIV (PLWH), there was a higher prevalence of detectable viremia in people reporting current smoking compared to people who reported never smoking (28.8% vs. 16.6%; p-value < 0.001). Active TB was more prevalent in people who currently smoked than in people who never smoked (3.1% vs 1.3%, p < 0.001). In contrast, the prevalence of hypertension and diabetes mellitus were lower in people reporting current smoking than in people reporting never smoking (17.1% vs 26.0% (p < 0.001), and 2.5% vs 10.2% (p < 0.001), respectively). In sex-stratified multivariable analyses that were adjusted for potential confounding factors (including body mass index for the NCDs), the magnitude of differences in CD prevalence between people who currently smoked and people who never smoked decreased, whereas the lower prevalence of NCDs among people reporting current smoking persisted.

**Conclusions:**

In rural South Africa, smoking is associated with higher rates of active TB, and people with HIV who smoke have worse disease control. In contrast, hypertension and diabetes mellitus are less common in those who smoke. Interventions to screen for TB among those who smoke and to address smoking among people with HIV may be particularly impactful.

## Introduction

South Africa faces the challenge of co-occurring epidemics of communicable disease (CDs) and non-communicable diseases (NCDs) [1]. We have previously reported on the Vukuzazi study, which estimated the prevalence and overlap of four common and treatable CDs and NCDs (HIV, tuberculosis [TB], hypertension and diabetes mellitus) among individuals aged 15 years and older in a population in KwaZulu-Natal, South Africa [2]. In this cohort, at least one of these four conditions was present in 52.1% of the > 18,000 participants, and 11.8% had two or more active conditions.

Tobacco use is the leading preventable cause of death globally and disproportionately impacts the health of people in low and middle-income countries (LMICs), where 80% of people who use tobacco live [3]. In the United States of America, among people living with HIV (PLWH) on antiretroviral therapy, smoking is now expected to reduce life expectancy more than HIV itself [4]. Similarly, tobacco smoking has been associated with poor TB outcomes, including higher mortality [5, 6]. Tobacco smoking, like hypertension and diabetes mellitus, is a major risk factor for cardiovascular disease and death [7–9].

However, there are limited data about the intersection of tobacco use with chronic diseases in sub-Saharan Africa, where CDs, such as HIV and TB, and NCDs, such as hypertension and diabetes mellitus, are widely prevalent. We sought to understand the co-occurrence of tobacco smoking with CDs and NCDs in rural South Africa using data from the Vukuzazi cohort.

## Methods

### Study participants and setting

The Vukuzazi population-wide health screening study was conducted among people 15 years of age and older by the Africa Health Research Institute in uMkhanyakude district in northern KwaZulu-Natal, South Africa between 2018–2020. Most of the uMkhanyakude area is poor and rural, with several informal periurban settlements and a single urban township [10]. Details of the Vukuzazi study were published previously [11]. In brief, study measurements included HIV testing with an immunoassay and HIV-1 RNA viral load (in people with a positive immunoassay). TB screening was conducted using symptom screen and digital chest x-ray, and for people with abnormalities on either of these, sputum was collected for molecular (GeneXpert MTB/RIF) and microbiological (MGiT) testing [12]. Blood pressure and hemoglobin A1c were measured as indicators of hypertension and diabetes mellitus, respectively.

### Exposure variable

Our primary exposure of interest was self-reported tobacco smoking, categorized as current (current use of tobacco products, such as cigarettes, cigars, pipes, bidis, hukahs or tamakhus, within the one year prior to survey), former (ever smoked tobacco and quit smoking at least one year prior to survey) or never (never smoked any tobacco products in the past).

### Outcome variables

Our primary outcomes of interest were the presence of the following conditions: (1) HIV, defined as participants with a positive HIV immunoassay; (2) active TB, defined as on TB treatment or with newly diagnosed sputum-positive TB (GeneXpert MTB/RIF and/or MGiT culture positive); (3) hypertension, defined as self-reported diagnosis/treatment and/or by measurements of blood pressure (BP) (systolic BP ≥ 140 mm Hg and/or diastolic BP ≥ 90 mm Hg); and (4) diabetes mellitus, defined as self-reported diagnosis/treatment and/or hemoglobin A1c > 6.5% [11]. Among PLWH, we further defined whether individuals had detectable HIV viremia (≥ 400 copies/mL) or undetectable HIV viral load (< 400 copies/mL).

### Covariates

We included the following variables as covariates to account for potential confounding between smoking and disease prevalence: age, body mass index (BMI, categorized as < 18.5 kg/m^2^: underweight, 19–24.9 kg/m^2^: normal, 25–29.9 kg/m^2^: overweight, ≥ 30 kg/m^2^: obese), personal economic activity (employed, unemployed, or part-time), and household socio-economic status (SES, estimated through a principal components analysis of asset ownership) [13].

### Statistical analysis

To assess the relationship between smoking and CDs and NCDs, we calculated and compared the percent of participants with each disease, stratified by smoking status (current, former and never). Next, we utilized multivariable logistic regression for each of the outcomes. Based on published literature, we determined, *a priori*, candidate variables to be included in the models [2]. Body mass index (BMI) was added to the model for NCDs. Multivariable models were stratified by sex because of the extreme over-representation of males in the current smoking group. We used Stata Special Edition (version 17.1) program.

## Results

Of the 18,024 participants in the Vukuzazi cohort (68% female, median age 37 years [interquartile rage 23–56 years]), 1,301 (7.2%) reported current smoking, 150 (0.8%) reported former smoking and 16,573 (92%) reported never smoking. Of those who currently smoked, 1069 (82%) reported smoking cigarettes, 175 (13%) smoking cigars, 23 (1.8%) smoking pipes, 441 (33.9%) smoking bidis, and 37 (2.8%) smoking hukkah either daily or weekly. Table 1 shows the demographic characteristics of the participants by smoking status. Among people who reported current smoking, the median age was 37 years (interquartile rage 27–49 years) and the majority were male (n = 1,177; 90.5%). Compared to people reporting former or never smoking, people reporting current smoking were more likely to be male, younger, and more overweight or obese. People who reported former smoking were older, more likely to be unemployed, and more likely to be in the poorest household socio-economic status quintile compared to those who reported current or never smoking.

The percentage of participants with HIV was similarly high among the three smoking categories (current 34.6%, former 33.6%, never 33.9%; p-value = 0.879) (Table 2). However, among PLWH, detectable viremia was more common in people reporting current smoking compared to people reporting former or never smoking (28.8% vs. 12.0% vs. 16.6%; p-value < 0.001). The percentage of participants with active TB was significantly higher among people reporting current or former smoking (current, 3.1%; former, 4.7%) compared to people who reported never having smoked (1.3%, p-value < 0.001). In contrast, hypertension and diabetes mellitus were less common in people reporting current smoking (17.1% and 2.5%, respectively) than people reporting former smoking (31.5% and 14.8%) or never smoking (26.2% and 10.2%, p-values for both hypertension and diabetes mellitus were < 0.001). Stratification of these data by sex revealed that among males, the percentage of participants who had each of the conditions defined (HIV, HIV with detectable viremia, active TB, hypertension and diabetes mellitus) all differed significantly by smoking status (Table 3). This includes a significantly higher prevalence of HIV in males reporting current smoking compared with males reporting former or never smoking. In contrast, in females, there were no significant differences in CDs or NCDs by smoking status (Supplementary Table 1).

Multivariable regression models comparing percentage of participants with CDs and NCDs among people with current smoking and never smoking status, stratified by sex and adjusted for potential confounders (age and socio-economic status), are shown in Table 4. We did not include former smoking in the multivariable regression models since only 150 participants (0.8%) were in this category. In the multivariable model, we found no relationship between being HIV-positive and current smoking in either males or females (adjusted odds ratio [aOR] = 1.10, 95% confidence interval [CI]: 0.93–1.30, p = 0.272; and aOR = 1.25, 95% CI: 0.83–1.89, p = 0.278, respectively), nor did we find any relationship between viremic HIV and current smoking in either males or females (aOR = 0.85, 95% CI 0.63–1.15, p = 0.291; and aOR = 0.87, 95% CI 0.43–1.77, p = 0.698, respectively). In males, we found an increased odds of active TB among people reporting current smoking compared with never smoking (aOR = 1.95, 95% CI: 1.22–3.13, p = 0.006). Also in males, current smoking was associated with lower odds of hypertension (aOR = 0.67, ,95% CI: 0.54–0.83, p-value < 0.001) and diabetes mellitus (aOR = 0.38, 95% CI: 0.24–0.61, p-value < 0.001) compared with never smoking. In females, there was no increase in odds of active TB, hypertension or diabetes mellitus in people reporting current smoking compared with people reporting never smoking. To better understand the relationship between body weight, tobacco use and chronic disease, we included BMI in the multivariable regression (Supplementary Table 2). The direction of all relationships held true in this analysis.

## Discussion

In a large population-based cohort in rural South Africa, viremic HIV and active TB were more common, and hypertension and diabetes mellitus were less common, in people reporting current smoking compared to never smoking. In this setting, over 90% of people reporting current smoking were males. Multivariable analyses, adjusted for potential confounding factors, indicated that among males, current smoking was associated with increased odds of active TB and decreased odds of hypertension and diabetes mellitus compared to never smoking.

In this study, relatively few participants reported current smoking, compared to other studies in South Africa [14–16]. Smoking prevalence in previous studies in South Africa varies by province, with much higher prevalence in the Western Cape province [14]. Stanton and colleagues examined the relationship between tobacco use and quality of life among adults with depression in Western Cape who were receiving antiretroviral therapy for HIV, finding that about 24% of the sample reported current (i.e., daily or weekly) tobacco use, with higher rates of current use among men (48.1%) than women (15.5%) [14]. The percentage of current smoking in the Vukuzazi study were high relative to one study that assessed smoking among PLWH in Johannesburg [15] and slightly low relative to another study among PLWH in Klerksdorp [16]. Waweru and colleagues [15] found an overall smoking prevalence of 15% among PLWH in Johannesburg, again with higher prevalence in men (23.3%) than women (7.4%), whereas 52% of men living with HIV in the Klerksdorp sample were defined as currently smoking [15]. Differences in sociodemographic factors and regions may account for these discrepancies, as prevalence of cigarette smoking differs greatly by province, with the highest prevalence among men reported in the Northern Cape and the highest prevalence among women reported in the Western Cape [17].

Our sex-stratified unadjusted analysis demonstrated significantly higher prevalence of HIV (overall) and uncontrolled (viremic) HIV among men who were currently smoking at the time of the survey compared to men who had never smoked. The fact that this relationship did not persist after multivariable analysis suggests that differences in age and socio-economic status may explain this effect. Nonetheless, our results suggest that people who are currently smoking should receive extra attention for services to diagnose and treat HIV. In addition, all PLWH who smoke should receive additional attention to ensure adherence to ART.

In keeping with several studies, we found a strong association between smoking and active TB [5, 18, 19]. We found a strikingly high prevalence of active TB among people who currently smoke – 3% – and people with a history of former smoking—6%. Multivariable analysis demonstrated that in males the relationship between current smoking and active TB persisted when adjusted for age and socio-economic status. These results indicate that people currently smoking or with a history of smoking should be prioritized for TB screening in addition to tobacco cessation interventions.

Unexpectedly, our data showed lower prevalence of hypertension and diabetes mellitus among men reporting current smoking compared with those who had never smoked, and this relationship persisted after multivariable adjustments for age, socio-economic status and BMI. This contrasts with previous findings [20]. Our results may be explained by the lack of access to health care in our setting and poor health seeking behaviors in males in our setting as found in Uganda [21]. Unmeasured confounders, such as family history of hypertension or diabetes mellitus, dietary factors, alcohol use, and physical activity, or residual confounding by age, may have also influenced the relationship. There also might be a survivor bias, such that some who smoked with active hypertension or diabetes mellitus might have died.

The current study has several limitations. Firstly, the cross-sectional design prevents the drawing of causal inferences. Secondly, data were collected from a rural geographic area in a single province, limiting the generalizability of the findings to urban communities and other groups in South Africa. In addition, we excluded from the logistic regression models the small number of participants who reported former smoking, potentially masking other associations between smoking with CDs and NCDs that may be apparent with larger study sizes. Moreover, it is feasible that healthier individuals were more likely to move out of the surveillance area in recent times, thereby potentially biasing the rate of the health outcomes.

## Conclusions

In this population-based sample of rural residents of South Africa, we found higher prevalence of active TB and virologically uncontrolled HIV and lower prevalence of hypertension and diabetes mellitus in those reporting current smoking compared with those reporting former or never smoking. These associations require further exploration, including longitudinal studies, and targeted health screening and tobacco cessation interventions in South Africa to reduce the complications of HIV and TB. The higher prevalence (3–6%) of active TB among those reporting current or former smoking is particularly noteworthy and suggests that these populations be screened more frequently for TB in TB-endemic regions. Combining tobacco cessation interventions with TB screening and/or antiretroviral adherence interventions may provide important individual and public health benefits.

## Figures and Tables

**Table 1:** Participant characteristics, by smoking category (n = 18,041)

	Total	Current smoking	Former smoking	Never smoking	P-value
Characteristic (n, %)	(N = 18,024)	(N = 1,301)	(N = 150)	(N = 16,573)	
**Sex**					<0.001
Male	5,812 (32.2%)	1,177 (90.5%)	101 (67.3%)	4,522 (27.3%)	
Female	12,229 (67.8%)	124 (9.5%)	49 (32.7%)	12,051 (72.7%)	
**Age category at enrolment, years**					<0.001
15–44	10,970 (60.8%)	872 (67.0%)	58 (38.7%)	10,025 (60.5%)	
>45	7,071 (39.2%)	429 (33.0%)	92 (61.3%)	6,548 (39.5%)	
**BMI category** *					<0.001
Underweight	857 (4.8%)	136 (10.5%)	10 (6.8%)	711 (4.3%)	
Normal	7,053 (39.5%)	879 (68.4%)	69 (47.3%)	6,105 (37.2%)	
Overweight	4,048 (22.7%)	179 (13.9%)	33 (22.6%)	3,836 (23.4%)	
Obese	5,884 (33.0%)	92 (7.2%)	34 (23.3%)	5,758 (35.1%)	
**Personal economic activity**					<0.001
Employed	2,759 (18.0%)	281 (22.6%)	20 (14.8%)	2,457 (17.6%)	
Part-time	617 (4.0%)	79 (6.4%)	7 (5.2%)	531 (3.8%)	
Not employed	11,976 (78.0%)	884 (71.1%)	108 (80.0%)	10,973 (78.6%)	
**Household socio-economic status quintile**					0.031
1 (poorest)	2,685 (15.2%)	237 (18.5%)	30 (20.4%)	2,417 (14.9%)	
2	3,600 (20.3%)	268 (20.9%)	28 (19.0%)	3,298 (20.3%)	
3	3,856 (21.8%)	264 (20.6%)	30 (20.4%)	3,559 (21.9%)	
4	3,943 (22.3%)	269 (21.0%)	31 (21.1%)	3,638 (22.4%)	
5 (wealthiest)	3,615 (20.4%)	244 (19.0%)	28 (19.0%)	3,341 (20.6%)	

P-values shown are from Chi-Square tests for smoking category (including former smoking) with demographic characteristics. All values are frequencies are reported with percentages in parenthesis.

* BMI: Less than 18.5 = Underweight, 18.5 to 24.9 = Healthy, 25 to 29.9 = Overweight and 30 or higher = Obese.

**Table 2:** Communicable and non-communicable diseases by smoking category

VARIABLE	Total	Current smoking	Former smoking	Never smoking	
	(N = 18,024)	(N = 1,301)	(N = 150)	(N = 16,573)	P-value
**HIV ELISA result**					0.879
Positive	6,096 (34.0%)	448 (34.6%)	50 (33.6%)	5,598 (33.9%)	
Negative	11,853 (66.0%)	847 (65.4%)	99 (66.4%)	10,907 (66.1%)	
**Viral load, among those with HIV**					<0.001
<400 copies/mL	5,070 (82.5%)	321 (71.2%)	44 (88.0%)	4,705 (83.4%)	
>=400 copies/mL	1,074 (17.5%)	130 (28.8%)	6 (12.0%)	938 (16.6%)	
**Active tuberculosis**					<0.001
Yes	245 (1.5%)	38 (3.1%)	6 (4.7%)	201 (1.3%)	
No	16,549 (98.5%)	1,179 (96.9%)	123 (95.3%)	15,237 (98.7%)	
**Hypertension**					<0.001
Yes	4,603 (25.6%)	222 (17.1%)	47 (31.5%)	4,334 (26.2%)	
No	13,373 (74.4%)	1,076 (82.9%)	102 (68.5%)	12,195 (73.8%)	
**Diabetes mellitus**					<0.001
Yes	1,737 (9.7%)	32 (2.5%)	22 (14.8%)	1,683 (10.2%)	
No	16,216 (90.3%)	1,262 (97.5%)	127 (85.2%)	14,827 (89.8%)	

P-values shown are from Chi-Square tests for smoking category (including former smoking) with communicable diseases and non-communicable diseases. All Values are frequencies reported with percentages in parentheses

**Table 3:** Communicable and non-communicable diseases by smoking category for males

VARIABLE	Total	Current smoker	Former smoking	Never smoker	
	(N = 5,800)	(N = 1,177)	(N = 101)	(N = 4,522)	P-value
**HIV ELISA result**					<0.001
Positive	1,406 (24.4%)	391 (33.4%)	28 (27.7%)	987 (21.9%)	
Negative	4,365 (75.6%)	780 (66.6%)	73 (72.3%)	3,512 (78.1%)	
**Viral load category**					0.012
<400 copies/mL	1,051 (73.7%)	275 (69.8%)	26 (92.9%)	750 (74.7%)	
>=400 copies/mL	375 (26.3%)	119 (30.2%)	2 (7.1%)	254 (25.3%)	
**Active tuberculosis**					0.001
Yes	111 (2.1%)	35 (3.2%)	5 (5.5%)	71 (1.7%)	
No	5,246 (97.9%)	1,067 (96.8%)	86 (94.5%)	4,085 (98.3%)	
**Hypertension**					0.001
Yes	1,042 (18.0%)	189 (16.1%)	31 (31.0%)	822 (18.2%)	
No	4,743 (82.0%)	986 (83.9%)	69 (69.0%)	3,688 (81.8%)	
**Diabetes mellitus**					<0.001
Yes	315 (5.5%)	24 (2.1%)	13 (12.9%)	278 (6.2%)	
No	5,455 (94.5%)	1,146 (97.9%)	88 (87.1%)	4,221 (93.8%)	

P-values shown are from Chi-Square tests for smoking category (including former smoking) with communicable diseases and non-communicable diseases. All values are frequencies reported with percentages in parentheses

**Table 4:** Association between communicable and non-communicable diseases and smoking status, multivariable regression.

VARIABLE	MALES		FEMALES	
	Adjusted odds ratio (95% CI)	P-value	Adjusted odds ratio (95% CI)	P-value
**HIV (positive ELISA result)**				
Never smoking	Reference		Reference	
Current smoking	1.10 (0.93; 1.30)	0.272	1.25 (0.83; 1.89)	0.278
**Viral load < 400 copies/mL among those with HIV**				
Never smoking	Reference		Reference	
Current smoking	0.85 (0.63; 1.15)	0.291	0.87 (0.43; 1.77)	0.698
**Active tuberculosis**				
Never smoking	Reference		Reference	
Current smoking	1.95 (1.22; 3.13)	0.006	1.95 (0.58; 6.53)	0.280
**Hypertension**				
Never smoking	Reference		Reference	
Current smoking	0.67 (0.54; 0.83)	<0.001	0.80 (0.49; 1.30)	0.366
**Diabetes mellitus**				
Never smoking	Reference		Reference	
Current smoking	0.38 (0.24; 0.61)	<0.001	0.63 (0.30; 1.34)	0.227

For each outcome variable (HIV, viral load < 400 copies/mL, tuberculosis, hypertension, and diabetes mellitus), we ran separate multivariable logistic regression models, adjusting for age and household socio-economic status. All logistic regression models excluded former smoking due to small numbers in the category. CI: Confidence Interval

## Data Availability

Data available in a publicly accessible repository (https://data.ahri.org/index.php/catalog/1006) which is managed in terms of AHRI’s Data Access Policy (https://www.ahri.org/)
